# Chronic allograft nephropathy

**DOI:** 10.1007/s00467-008-0869-z

**Published:** 2009-08-01

**Authors:** Jeffery T. Fletcher, Brian J. Nankivell, Stephen I. Alexander

**Affiliations:** 1 Department of Paediatrics, The University of Sydney, Nepean Clinical School, Nepean Hospital, Sydney, NSW Australia; 2 Department of Renal Medicine, The University of Sydney, Western Clinical School, Westmead Hospital, Sydney, NSW Australia; 3 Centre for Kidney Research, The Children’s Hospital at Westmead, The University of Sydney, Western Clinical School, Westmead Hospital, Sydney, NSW Australia; 4 Centre for Kidney Research, The Children’s Hospital at Westmead, Lock Bag 4001, Westmead, 2145 NSW Sydney, Australia

**Keywords:** Chronic allograft nephropathy, CNI, Rejection, Kidney transplantation

## Abstract

Chronic allograft nephropathy (CAN) is the leading cause of renal allograft loss in paediatric renal transplant recipients. CAN is the result of immunological and nonimmunological injury, including acute rejection episodes, hypoperfusion, ischaemia reperfusion, calcineurin toxicity, infection and recurrent disease. The development of CAN is often insidious and may be preceded by subclinical rejection in a well-functioning allograft. Classification of CAN is histological using the Banff classification of renal allograft pathology with classic findings of interstitial fibrosis, tubular atrophy, glomerulosclerosis, fibrointimal hyperplasia and arteriolar hyalinosis. Although improvement in immunosuppression has led to greater 1-year graft survival rates, chronic graft loss remains relatively unchanged and opportunistic infectious complications remain a problem. Protocol biopsy monitoring is not current practice in paediatric transplantation for CAN monitoring but may have a place if new treatment options become available. Newer immunosuppression regimens, closer monitoring of the renal allograft and management of subclinical rejection may lead to reduced immune injury leading to CAN in the paediatric population but must be weighed against the risk of increased immunosuppression and calcineurin inhibitor nephrotoxicity.

## Introduction

Chronic allograft nephropathy (CAN) is a histopathological diagnosis used to denote features of chronic interstitial fibrosis and tubular atrophy within the renal allograft. It remains the most common cause of graft dysfunction and loss in children following renal transplantation. The incidence and pathological processes involved in chronic graft loss in children with renal transplants appear similar to those found in adult renal transplants. The term CAN replaced chronic allograft rejection. Previously, chronic allograft rejection was considered the major aetiological factor for chronic graft loss, as features of cellular inflammatory immune infiltrates, identified on kidney biopsies, were suggestive of injury from immunological changes within the graft. This classification changed with the implementation of the Banff 97 working classification of renal allograft pathology criteria [1], which included features of the Chronic Allograft Damage Index [2] and Co-operative Clinical Trials in Transplantation systems. This led to the standardisation and semiquantification of these lesions. It was revised again in 2003 with the addition of C4d, a cleavage product of activated complement factor 4, as a marker of antibody-mediated injury [1, 3] and again in 2007 when the nonspecific deficits of sclerosing CAN was replaced by the more accurate term interstitial fibrosis not otherwise specified. These criteria all use histopathological markers of renal transplant injury to define the level and severity of allograft damage and selected specific features that imply a specific diagnosis.

## Histopathology

**Fig. 1:**
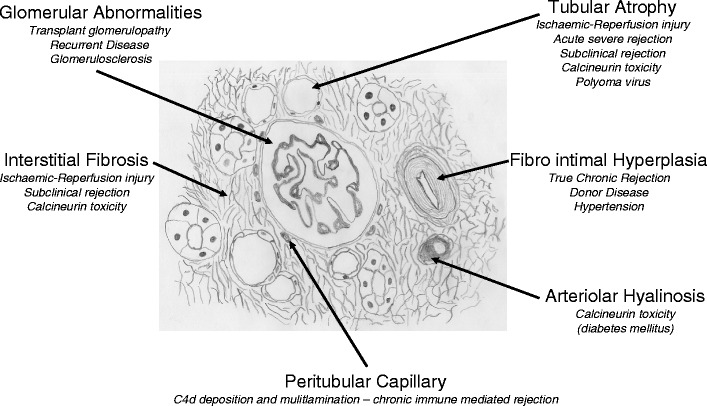
Schematic illustration of biopsy of a renal allograft showing histopathological features characteristic of chronic allograft nephropathy (CAN).* Italics* indicate potential precipitating factors for CAN associated with the areas they specifically target. Reprinted with permission from [51]

**Fig. 2:**
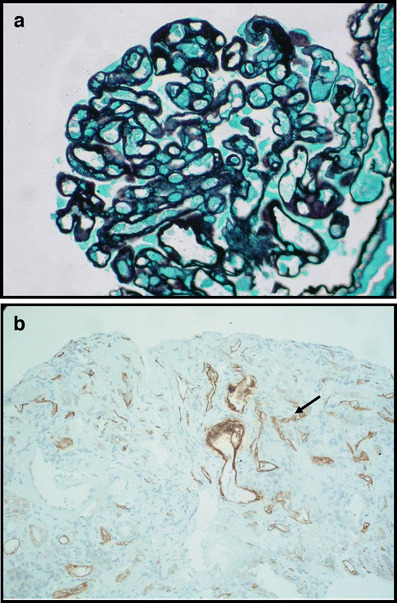
**a** Renal allograft biopsy (*silver staining*) showing features of transplant glomerulopathy with evidence of “double contours” in capillary loops, mesangial proliferation and matrix expansion and basement membrane thickening. **b** Renal allograft biopsy from a child with chronic allograft nephropathy (CAN) showing C4d deposition (*in brown*) in peritubular capillaries consistent with humoral-mediated rejection

The histological features that define CAN in the kidney transplant allograft include interstitial fibrosis and tubular atrophy, as mentioned above, as well as features of glomerulosclerosis and fibrointimal hyperplasia (Fig. 1). CAN is graded as mild, moderate or severe based on the severity of chronic interstitial fibrosis and tubular atrophy and the area of cortex affected in the biopsy specimen. Interstitial fibrosis, denoted as ci, is scored by the area fibrosed and ranges from mild (ci1 6–25%) to severe (ci3 >50%). Tubular atrophy refers to the loss of tubular height and increased luminal size of the tubules and is denoted as ct (ct0–ct3). Tubular atrophy and interstitial fibrosis are often nonspecific by themselves. Chronic transplant glomerulopathy refers to the thickening of the glomeruli and is quantified by the percentage of glomeruli developing “double contours” of peripheral capillary loops and is denoted as cg (cg0–cg3) (Fig. 2a). Arteriolar hyalinosis, as suggested by the term, denotes thickening of arterioles within the kidney based on the amount of periodic-acid-Schiff-positive hyalinosis and is denoted as ah (ah0–ah3), often implying calcineurin inhibitor (CNI) nephropathy. More in-depth quantification of all of these criteria is readily available [1].

The addition of C4d staining to the Banff criteria in 2003 has allowed for the supportive diagnosis of chronic antibody-mediated rejection. C4d is a positive marker of complement activation, implying the presence of antidonor antibodies and hence antibody-mediated rejection. C4d is released on binding to antibody. These antibodies bind to endothelial cells in glomerular and peritubular capillaries, suggesting antibody deposition [4, 5] (Fig. 2b) and prompting the clinician to request donor-specific antibody testing. C4d staining is regarded as positive or negative, and its position within the biopsy is recorded and graded by type, as acute tubular necrosis (ATN)-like, capillary or arterial [6]. C4d is gaining increasing routine use in the paediatric transplant population [7] and has a role in acute rejection, early unexplained primary graft nonfunction and chronic dysfunction, especially where transplant glomerulopathy is present [5].

The evidence for chronic allograft nephropathy as the leading cause for progressive renal failure and graft loss is supported by both transplant registry and protocol biopsy data. Graft loss secondary to the progressive development of CAN has consistently been recorded within the European, North American (NAPRTCS) [8] and Australian–New Zealand (ANZDATA) transplantation registries. Although histological confirmation of CAN by biopsy is variable, reports from all databases show progressive transplant loss attributable to CAN continuing to the present day despite improved changes to immunosuppression regimens. Cohort studies using protocol biopsies performed in child and adult transplant recipients from day of transplant to 10 years posttransplantation consistently demonstrate the evolution and progression of CAN [9–16]. Larger studies have helped identify aetiological factors involved in chronic graft injury. In particular, the 10-year protocol biopsy study on adult patients with kidney–pancreas transplants defined the occurrence of severe rejection, of subclinical rejection and in some cases true chronic rejection, as evidenced by tubulointerstitial damage, with increasing evidence of progressive nephropathy from CNIs. Histological lesions of grade 1 CAN present in up to 94.2% of adult patients at 1 year posttransplant [11, 12], and grades progressively worsen up to 10 years. Protocol biopsy studies in children demonstrate similar features of CAN [17–23]. The most recent of these, with 245 biopsies in 81 children over 2 years, found subclinical rejection (SCR) at 3 months and progressive changes to CAN over 24 months [20].

## Mechanisms of injury

CAN results from the accumulation of immune and nonimmune insults to the kidney. Numerous aetiological factors have been implicated, including immunological factors, drug toxicity, donor disease, recurrent disease and infections. The immune mechanisms of acute rejection involve predominantly direct antigen presentation, whereas previous episodes of acute cellular rejection (ACR) and acute humoral rejection (AHR) may leave residual injury that predisposes to CAN. However, there is increasing evidence that chronic immune injury may involve donor-derived peptides being indirectly presented by host antigen-presenting cells leading to immune sensitisation and damage. Other pathways may include chronic humoral rejection with the presence of C4d, glomerular changes and peritubular multilammination by electron microscopy [4, 5]. Newer issues include certain innate pathways using the major histocompatibility complex (MHC) class-I-related chain A (MICA) set of antigens, which have now been implicated in acute rejection and may play a role in CAN [24, 25]. Recent evidence suggesting the reduction in regulatory T-cell (Treg) numbers, and inhibition of their function by CNIs [26, 27] in human transplantation and animal data suggesting that Tregs may help to limit CAN [28], may imply a protective role for Tregs in CAN development.

## Aetiological factors of CAN

### Donor factors

There are a number of donor factors associated with CAN, which include: (1) donor age where extremes of donor ages do poorly; (2) preexisting disease or injury to the donor; (3) degree of HLA mismatch; (4) organ-specific issues such as prolonged cold ischaemia time and delayed graft function; (5) male donors have better outcomes than female donors probably related to organ size; and (6) living related and living unrelated donors have better outcomes than deceased donors related to ischaemia reperfusion injury.

### Recipient factors associated with CAN

In the recipient, age is again a risk factor, with younger children being at greater risk for graft loss, although NAPRTCS data suggest adolescence is associated with increased graft loss largely from noncompliance [29]. Preexisting disease can affect long-term outcome, with disease recurrence occurring in conditions such as focal segmental glomerulosclerosis, atypical haemolytic uraemic syndrome, congenital nephrotic syndrome and immunoglobulin A (IgA) nephropathy. Prior sensitisation from previous transplants, blood transfusions and race, particularly African American recipients, are associated with a poorer outcome. Small recipient size transplanted with large adult-sized renal allografts—that is, donor–recipient size discrepancy—has long been considered a nonimmune risk factor for graft failure and CAN. Recent evidence in a protocol biopsy study of such recipients on CNI immunosuppression but no clinical episodes of rejection identified SCR at 3 months and the progression of histological changes of CAN to 24 months [20]. Whereas recognition of the need to maintain early graft perfusion in small recipients has led to a decline in early graft loss from thrombosis, protocol biopsy data suggests that ongoing chronic injury can result from hypoperfusion of a large kidney in a small child. Immune matching plays a role, with full HLA matching reducing the risk of graft loss. In paediatrics, the presentation of de novo antigens to children with recessive or X linked disease receiving a kidney is an interesting concept suggested by the protocol biopsies of children with congenital nephrotic syndrome, which show a high frequency of glomerulosclerosis [30].

### Calcineurin inhibitors

The association of specific CNI changes leading to transplant damage has been related to duration of exposure and dose in protocol biopsy studies [11, 12]. Calcineurin use in children, although improving first-year graft survival, has not led to a marked improvement in long-term graft loss [8]. The one randomised study of continued cyclosporine versus early cyclosporine withdrawal with a 15-year follow-up suggested that whereas CNIs provide a greater benefit initially, there is then a greater progressive decline in graft function related to CNI toxicity [31]. The mechanism of CNI toxicity may involve the induction of transforming growth factor-beta (TGF-β) in renal tubular cells by CNI, leading to progressive fibrosis [32].

Specific histological features of CNI toxicity including striped fibrosis and arteriolar hyalinosis are identified as early as 3 months posttransplant and are one of the major contributors to the development of CAN, with 100% of adult transplant recipients showing some histological evidence of CNI toxicity by 10 years posttransplant [11]. The only paediatric CNI-free regimen, the CN-01 study [23] involving induction with sirolimus, anti-CD25 monoclonal antibody and maintenance prednisone, sirolimus and mycophenolate mofetil (MMF), identified no evidence of interstitial fibrosis and tubular atrophy in 12-month protocol biopsies of children who did not experience rejection at 12 months. CNI reduction studies in children maintained on MMF [33, 34] or MMF introduction in children with histological CAN [35–37] showed renal function stabilisation but have not yet reported histological data showing the slowed progression of CAN and further long-term follow-up [33]. However, study protocols that minimise immunosuppression may result in higher rates of SCR and ACR and progression to CAN [19], whereas those that use greater initial immunosuppression run the risk of infectious complications and posttransplant lymphoproliferative disease (PTLD) [23].

### Infection

With the new powerful immunosuppressive regimens, infectious complications after transplantation are the leading cause of paediatric hospital admission [38]. The major viruses leading to admission and of increasing concern as precipitating factors for chronic renal injury are Epstein-Barr virus (EBV), cytomegalovirus (CMV) and BK virus (BKV). As the incidence of positive donors to negative recipients increases in paediatric transplant recipients, the risk of viral reactivation in the graft and primary infection is likely to increase. BKV nephropathy, for example, can lead to graft loss in 50% of patients within 2 years [39], though improved antiviral prophylaxis and treatment for posttransplant viral infections—such as ganciclovir for CMV and leflunomide, ciprofloxacin and cidofovir for BKV nephropathy—have improved acute disease outcome. However, little data are available on the efficacy of their use in reducing the risk of CAN. EBV-associated PTLD managed by reduction in immunosuppression and anti-CD20 monoclonal antibody (rituximab) has improved patient and disease outcome [40], but progression to CAN is more rapid following EBV disease.

### Compliance

Adolescent transplant recipients may be noncompliant, and children are at greater risks of variations in the immunosuppression related to this [41]. Compliance and kidney function has been evaluated in studies, including a rigorous study from Minnesota, USA, that demonstrated that noncompliant patients lose grafts more readily [29], most likely due to the initiation of subclinical chronic immune injury during a noncompliant period leading to CAN development and permanent nephron loss.

### Subclinical rejection

Subclinical rejection is the histological presence of immunoreactive cells within the kidney allograft suggestive of acute rejection without clinical deterioration of renal function; i.e. renal function remains stable. Untreated SCR can lead to the development of overt ACR with graft dysfunction or may smoulder on to eventual CAN. The incidence of subclinical inflammatory infiltrates of 35–53% [18, 42] in protocol biopsies at 3 months post paediatric kidney transplants are similar to adult reports [11], and both groups of patients show reduction in SCR rates by 12–18 months [11, 42].

In paediatric transplant recipients with large kidney grafts and increased early GFR proportionate to size, nonimmune rejection processes may be advanced before functional changes are detected by serum creatinine measurement [20], leading to ongoing debate on the need for protocol biopsies in children, which is discussed below. However, the rates of SCR are lower with modern immunosuppression, so that it is unclear whether the advantage of increasing immunosuppressive treatment is outweighed by infectious risks.

### Protocol biopsies

Protocol biopsies can be performed after transplantation to detect early subclinical rejection, signs of CAN or CNI nephropathy and to monitor graft anatomy in children with stable renal function. Although not routine clinical practice in paediatric renal transplantation, they have been performed as part of a number of clinical studies [43]. Recent reports confirm the safety of this procedure [44]. The incidence of CAN in paediatric protocol biopsy cohorts ranged from 30% to 100% by 2 years posttransplant [17–19, 21, 22], which supports a potential role in routine practice that may influence treatment. SCR treatment in adults and children involving high-dose pulse corticosteroids is supported yet not proven in cohort studies showing the reduction of histological changes in CAN [45] or its stabilisation [46–48]. The benefits of treating SCR, however, and the strengthening of immunosuppression that is usually required, need to be weighed against the increased risk of primary infection, viral reactivation such as CMV, BK and EBV, and PTLD.

### Newer ways of identifying and classification of CAN

Improvements in the use of molecular genetics through gene expression arrays and an increased ability to identify variability in genes by genome-wide or molecular pathway-specific arrays suggest that we may be able to move to a molecular classification of allograft damage at some point. It may be that genetic differences in both recipient and donor are important, and identifying these factors in both may allow us to view not just disease progression but possibly predict and modify risk. Despite the variability in results from genomics, proteomics and expression arrays, a recent review of the Banff classification (Banff 05) led to support for consideration of a broader range of aetiologies and the potential for a genomics-supported classification [49]. This is an evolving process to provide more consistent evaluation of renal biopsies leading to improved diagnostic and therapeutic outcomes. [50].

### New therapies

A number of new agents have been tested in adults and children, including sirolimus, everolimus, FTY720 and protocols that use high levels of immunosuppression early to reduce long-term immunosuppression either by discontinuing steroids or discontinuing CNIs using anti-interleukin-2R (IL-2R) antibodies or immune-depleting antibodies such as Campath. Other new agents include the new CTLA4-Ig antibody belatacept, which has been used successfully in place of CNIs in adult studies of kidney transplantation [51] and the Janus kinase (JAK) inhibitors.

Success of standard treatment limits the ability to assess new agents, and the lack of good surrogate markers for transplant damage make evaluation of the different regimes difficult. There are also a number of new agents under development to prevent fibrosis, such as bone morphologic protein-7 (BMP-7) that may limit nonimmune renal injury and may have a place in renal transplants to extend survival.

In summary, CAN is a major cause of paediatric graft loss. There are both donor and recipient causes for this, and it is likely that it reflects a combination of both immune and nonimmune injury occurring cumulatively over time. Histological features of CAN and specific features that suggest the underlying aetiology are under regular revision. Protocol biopsies in children and management of SCR or early CAN with immunosuppression adjustment may potentially slow CAN progression but must be weighed against the more general risks of over- or underimmunosuppression.

## Questions

### (Answers appear after the reference list.)

1. Which of the following is not a histopathological feature of CAN?
  - Interstitial fibrosis
  - Tubular atrophy
  - Glomerulosclerosis
  - Striped fibrosis
  - Fibrointimal hyperplasia
2. Which of the following is not an infection that has been shown to increase progression to CAN?
  - Cytomegalovirus
  - Epstein-Barr Virus
  - Coxsackie virus (enterovirus)
  - Polyoma virus
  - BK virus
3. Calcineurin toxicity induces which one of the following to potentiate renal fibrosis and eventual progression to CAN?
  - TNF-α
  - VEGF
  - TGF-β
  - STAT 4
  - STAT 6
4. The addition of which one of the following leads to the improvement in diagnosing humoral-mediated rejection on renal biopsy?
  - C4d
  - C3
  - C4
  - C50
  - C1q
5. Donor factors associated with increased risk for the development of CAN include all of the following except?
  - Young age
  - Male sex
  - HLA mismatch
  - Large allograft size in small recipients
  - Prolonged cold ischaemia time
6. Subclinical rejection (SCR) is diagnosed by:
  - Clinically by a raised creatinine
  - Identification of molecular markers in the urine
  - Number of T cells present in the urine assessed by flow cytometry
  - Crystal ball
  - Histopathologically with stable renal function

### Answers

1. d
2. c
3. c
4. a
5. b
6. e

## Abbreviations

ACR: acute cellular rejection
AHR: acute humoral rejection
ANZDATA: Australian and New Zealand Dialysis and Transplantation Association
BKV: BK virus
CAN: chronic allograft nephropathy
CMV: cytomegalovirus
CNI: calcineurin inhibitors
EBV: Epstein-Barr virus
MMF: mycophenolate mofetil
NAPRTCS: North American Pediatric Renal Trials and Collaborative Studies
OKT3: orthoclone OKT3 (muromonab-CD3)
PTLD: posttransplant lymphoproliferative disease
SCR: subclinical rejection
Treg: regulatory T cells
TGF-β: transforming growth factor-beta
